# Vascularization converts the lineage fate of bone mesenchymal stem cells to endothelial cells in tissue-engineered bone grafts by modulating FGF2-RhoA/ROCK signaling

**DOI:** 10.1038/s41419-018-0999-6

**Published:** 2018-09-20

**Authors:** Donglin Li, Pengzhen Cheng, Huijie Jiang, Tianqing Cao, Jimeng Wang, Yi Gao, Yangjing Lin, Chunmei Wang, Shuaishuai Zhang, Junqin Li, Bin Liu, Yue Song, Liu Yang, Guoxian Pei

**Affiliations:** 1 Institute of Orthopedic Surgery, Xijing Hospital, Fourth Military Medical University, Xi’an, 710032 People’s Republic of China; 2 The 463th Hospital of People’s Liberation Army, Shenyang, 110042 People’s Republic of China; 3 Department of Orthopaedics, First Affiliated Hospital, Chengdu Medical College, Chengdu, 610500 People’s Republic of China

## Abstract

The prevascularization of tissue-engineered bone grafts (TEBGs) has been shown to accelerate capillary vessel ingrowth in bone defect remodeling and to enhance new bone formation. However, the exact mechanisms behind this positive effect remain unknown. Here, we report that basic fibroblast growth factor (FGF2)-Ras homolog gene family member A (RhoA)/Rho-associated protein kinase (ROCK) signaling functions as a molecular switch to regulate the lineage fate of bone mesenchymal stem cells (BMSCs) and that prevascularization promotes the cell fate switch, which contributes to increased bone regeneration with the use of prevascularized TEBGs compared with control TEBGs. Prevascularized TEBGs enhanced the in vivo endothelial differentiation of BMSCs by inhibiting RhoA/ROCK signaling. In vitro data more clearly showed that BMSCs differentiated into von Willebrand factor (vWF)-positive endothelial cells, and FGF2-induced inhibition of RhoA/ROCK signaling played a key role. Our novel findings uncovered a new mechanism that stimulates the increased vascularization of engineered bone and enhanced regeneration by promoting the endothelial differentiation of BMSCs implanted in TEBGs. These results offer a new molecular target to regulate TEBG-induced bone regeneration.

## Introduction

Large segmental bone defects caused by severe trauma and pathological fractures usually fail to heal naturally due to limited self-repairing capabilities^1,2^. To achieve perfect bone regeneration, bone grafts, including autografts and allografts, are applied to fill segmental defects^3,4^. In the past two decades, tissue-engineered bone graft (TEBG) techniques have offered a promising alternative therapy for large bone defects without side effects compared with traditional therapies^5–10^. TEBGs are generated by seeding bone mesenchymal stem cells (BMSCs) into scaffolds for in vivo transplantation^11^.

Vascularization is a crucial in vivo process in TEBG-mediated regeneration of large segmental bone defects. Usually, spontaneous vascularization results from an inflammatory response that occurs in the peripheral region of the scaffold, where the vascular ingrowth is limited to several tenths of micrometers per day^12^. This is too slow to provide enough nutrients for cells in the central region of the TEBG^13^. Therefore, regeneration of neovessels at an early stage after TEBG implantation is a major hurdle to overcome in achieving satisfactory healing^14,15^.

During the past few decades, several strategies to improve the vascularization process of TEBG were reported^16^. These strategies include modification of scaffold designs, supply of angiogenic factors, and surgical prevascularization^17–19^. Compared with other approaches, surgical prevascularization, including periosteal flap coverage, arteriovenous loop and vascular bundle insertion, demonstrates advantages to provide a stable and instantaneous perfusion, which dramatically decreases the time required for capillary ingrowth^20–23^. In our previous study, TEBGs were prevascularized by inserting femoral vascular bundles then implanted to treat large bone defects. The results indicated that the prevascularized TEBG group had a significantly higher volume of regenerated bone and new vessels^16,24,25^. However, the cellular and molecular mechanisms of accelerated bone regeneration after prevascularized TEBG implantation remain elusive.

In the present study, we delineated a molecular mechanism by which the FGF2- RhoA/ROCK signaling pathway regulates BMSCs fate in TEBGs.

FGF2 is considered a differentiation inducer and regulatory factor in stem cell research. It is upregulated in response to inflammatory stimuli^26^. According to Wang et al.^27^, the supplementation of stem cell culture medium with FGF2 alters the morphology and enhances the tri-lineage differentiation capacity of giant panda BMSCs. Morphological changes have been shown to affect the early commitment of pluripotent BMSCs to the adipose versus osteoblastic lineage via modulation of RhoA activity^28^. FGF2 has previously been suggested to modulate cytoplasmic RhoA/ROCK signaling^29,30^. Therefore, the aim of this study was to investigate the role of vascularization in tissue-engineered bone grafts and to determine whether the FGF2-mediated activation of the RhoA/ROCK signaling pathway induced BMSCs differentiation.

Our results showed that BMSCs differentiated into endothelial-like cells when co-cultured with endothelial cells, and this cell fate change was mediated by FGF2 via RhoA/ROCK signaling pathway modulation. These findings uncover a novel mechanism that explains the increase in vascularized bone regeneration by enhancing the endothelial differentiation of seeding BMSCs in TEBGs, and the results offer a new molecular target to regulate TEBG-related bone regeneration.

## Results

### Regeneration following the establishment of the large bone defect model in the rat femur and implantation of TEBGs

**Fig. 1:**
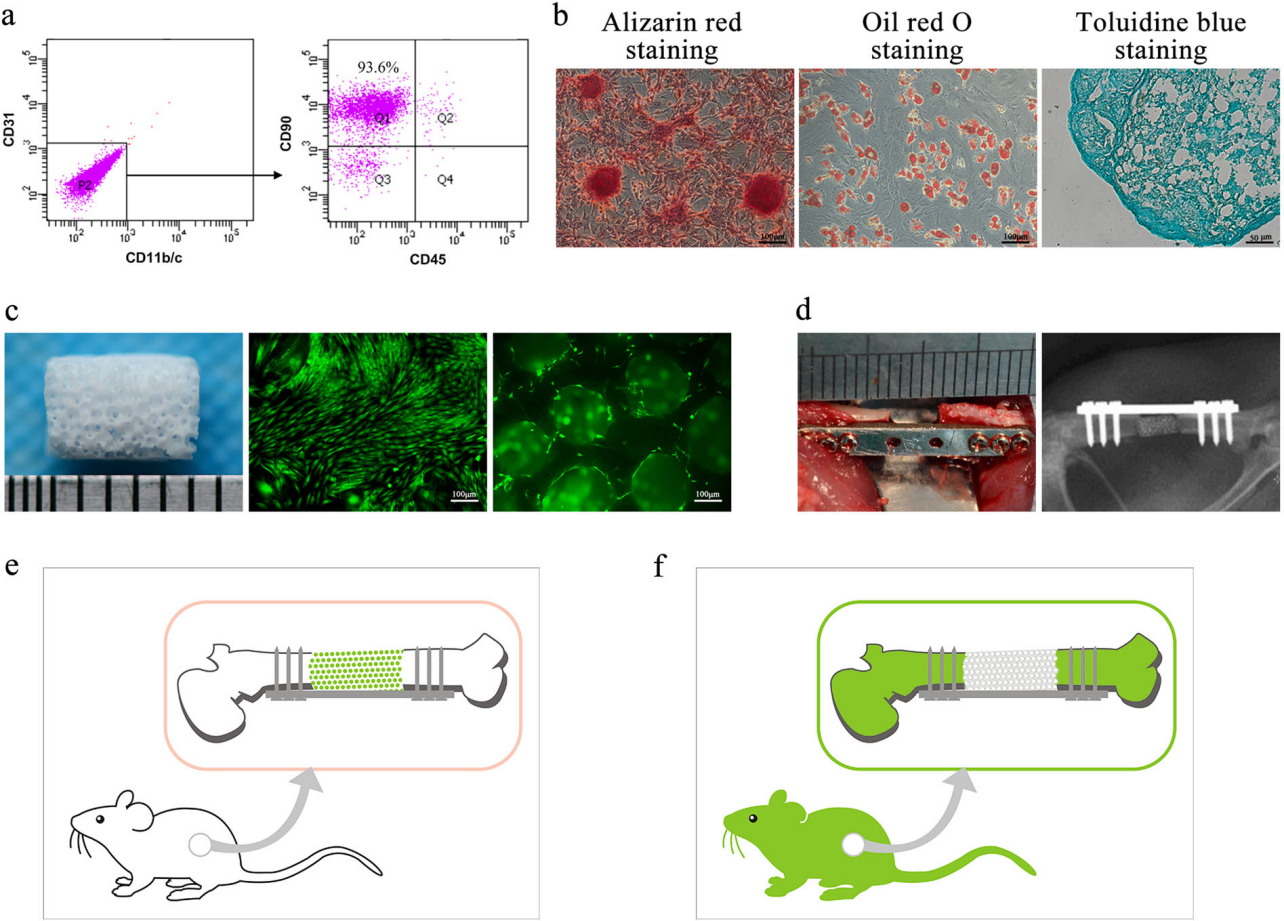
The large bone defect model in rat femur. Third-passage BMSCs were analyzed by flow cytometry. Most cells were CD31^-^ and CD11b/c^-^ (**a**, left panel), and a further representative image shows that the percentage of the CD31^-^, CD11b/c^-^ and CD45^−^ cells that express CD90 was 93.6% (**a**, right panel). **b** Multi-lineage differentiation assay of rat BMSCs: osteogenic, adipogenic and chondrogenic differentiation. **c** β-TCP scaffolds (5 mm in height and 4 mm in diameter, 70% porosity, 400 µm pore diameter) were incubated with GFP^+^ BMSCs for a week. **d** The femur bone defect was made in the left femur and fixed by internal fixation. After surgery, all rats were examined using X-ray imaging to confirm that the model was successful. **e**, **f** Two types of femur defect models were used: WT rats matched with GFP^+^ BMSCs-TEBGs and GFP^+^ rats matched with WT BMSCs-TEBGs

We characterized third-passage BMSCs by a flow cytometry method (FCM) analysis and multilineage induction. The FCM analysis revealed that 93.6% of the cells were CD31^−^CD11b/c^-^CD90^+^CD45^−^ (Fig. 1a). After 3 weeks of induction with the appropriate media, these BMSCs differentiated into osteoblasts, as shown by Alizarin Red S-positive staining, adipocytes, as shown by Oil Red O-positive staining, and chondrocytes, as shown by Toluidine Blue-positive staining (Fig. 1b).

A 5-mm customized TEBG scaffold was designed and processed with a slot using β-TCP. After infiltrating the β-TCP scaffolds with culture medium containing BMSCs for 2 h, the TEBGs were cultured in culture medium for 1 week. Using the same method, green fluorescent protein (GFP^+^) BMSCs-TEBGs and Wild-type (WT) BMSCs-TEBGs were respectively generated by using different origins of BMSCs (Fig. 1c).

A 5-mm large bone defect repair model was established in the diaphysis of rat femurs^31^. Cultured TEBGs were implanted and fixed using self-designed internal fixation plates (Fig. 1d). GFP^+^ BMSCs were used to generate GFP^+^ BMSCs-TEBG for further implantation into WT rats to trace the in vivo fate of seeded BMSCs (Fig. 1e). In contrast, WT BMSCs were used to generate WT BMSCs-TEBG for further implantation into GFP^+^ rats to trace the host cells. In this case, the GFP^+^ cells in TEBGs were all obtained from the host rat (Fig. 1f).

### Prevascularization of TEBGs facilitated the regeneration of rat bone in the large defect

**Fig. 2:**
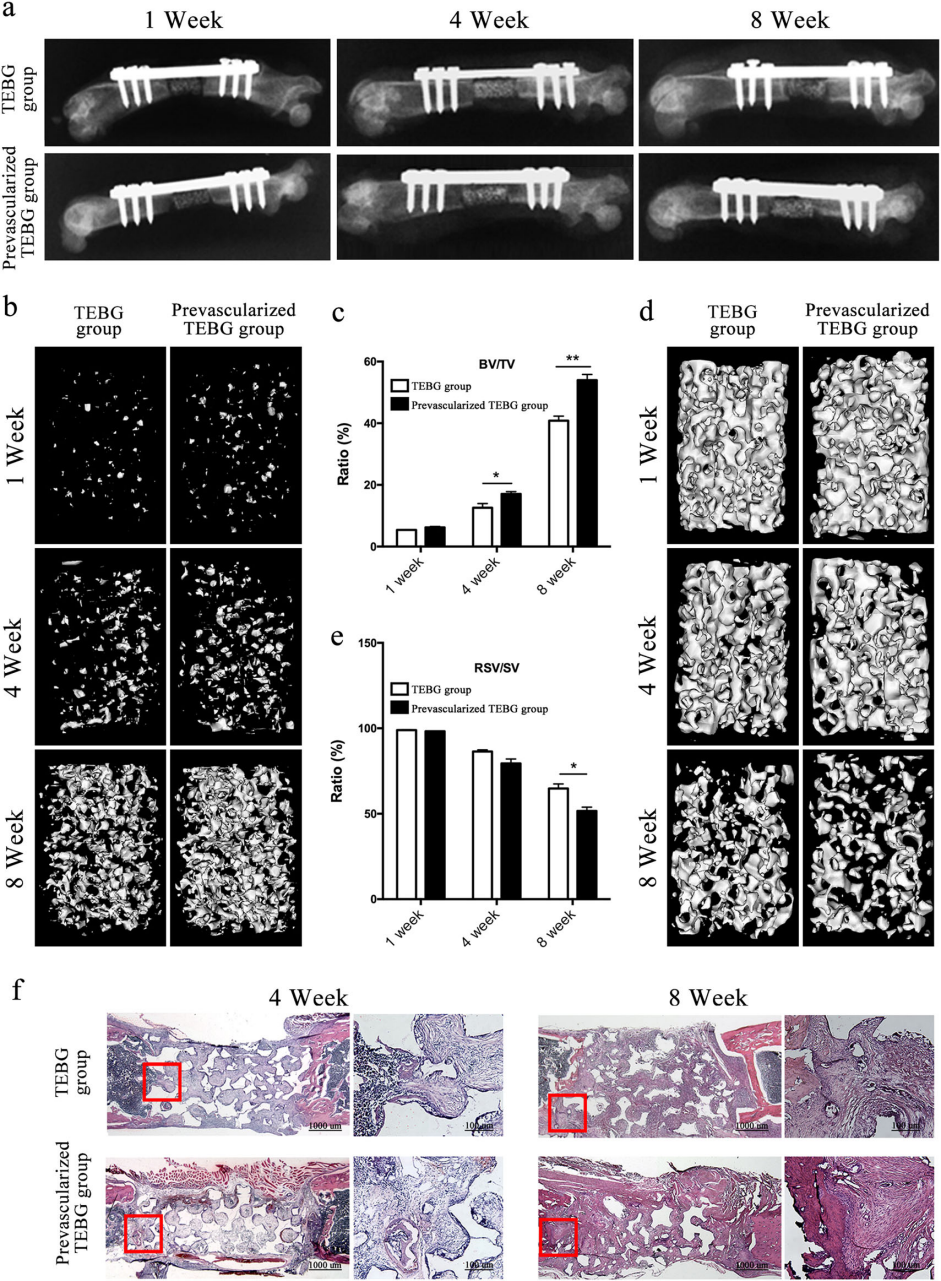
Prevascularized TEBGs promoted repair of bone defects. **a** After surgery, all rats were exposed to X-ray to affirm the model was successful. The images were collected at 1, 4 and 8 weeks after implantation. **b**, **d** Micro-CT 3D reconstruction images of the new bone formation and β-TCP matrix at 1, 4 and 8 weeks after surgery. **c** BV/TV was used to evaluate new bone formation. **e** RSV/SV was used to evaluate scaffold degradation. *n* = 10 samples per group. Data are represented as the mean ± s.e.m. *P < 0.05, **P < 0.01 as determined by Student’s t-tests. **f** H&E staining of rat femur sections at 4 and 8 weeks after the surgery

The internal fixation plates worked well for up to 8 weeks after the operation, as shown in the X-rays (Fig. 2a). And their microstructures and bone masses were evaluated by micro-CT scanning (Fig. 2b–d). By using different threshold values, both new bone formation and scaffold matrix were respectively evaluated. After 4 weeks, the prevascularized TEBG group displayed a significantly higher bone volume/total volume (BV/TV) value than the TEBG group (Fig. 2b, c). Additionally, significantly lower resident scaffold volume/scaffold total volume (RSV/SV) values were observed in the group with prevascularized TEBG scaffolds than in the TEBG group at 8 weeks (Fig. 2d, e).

Histological staining was performed to assess the osteointegration of the implants at 4 and 8 weeks after implantation (Fig. 2f). The inserted blood vessel was observed in the prevascularized TEBG group. After 4 and 8 weeks of regeneration, the scaffold pores within the TEBGs were filled by reticular tissue and osteoid matrix. Moreover, at 8 weeks, the scaffolds in the prevascularized TEBG group were degraded more, especially in the middle of the TEBGs.

### Prevascularization of TEBGs altered the endothelial and osteogenic differentiation of seeded cells and host cells

**Fig. 3:**
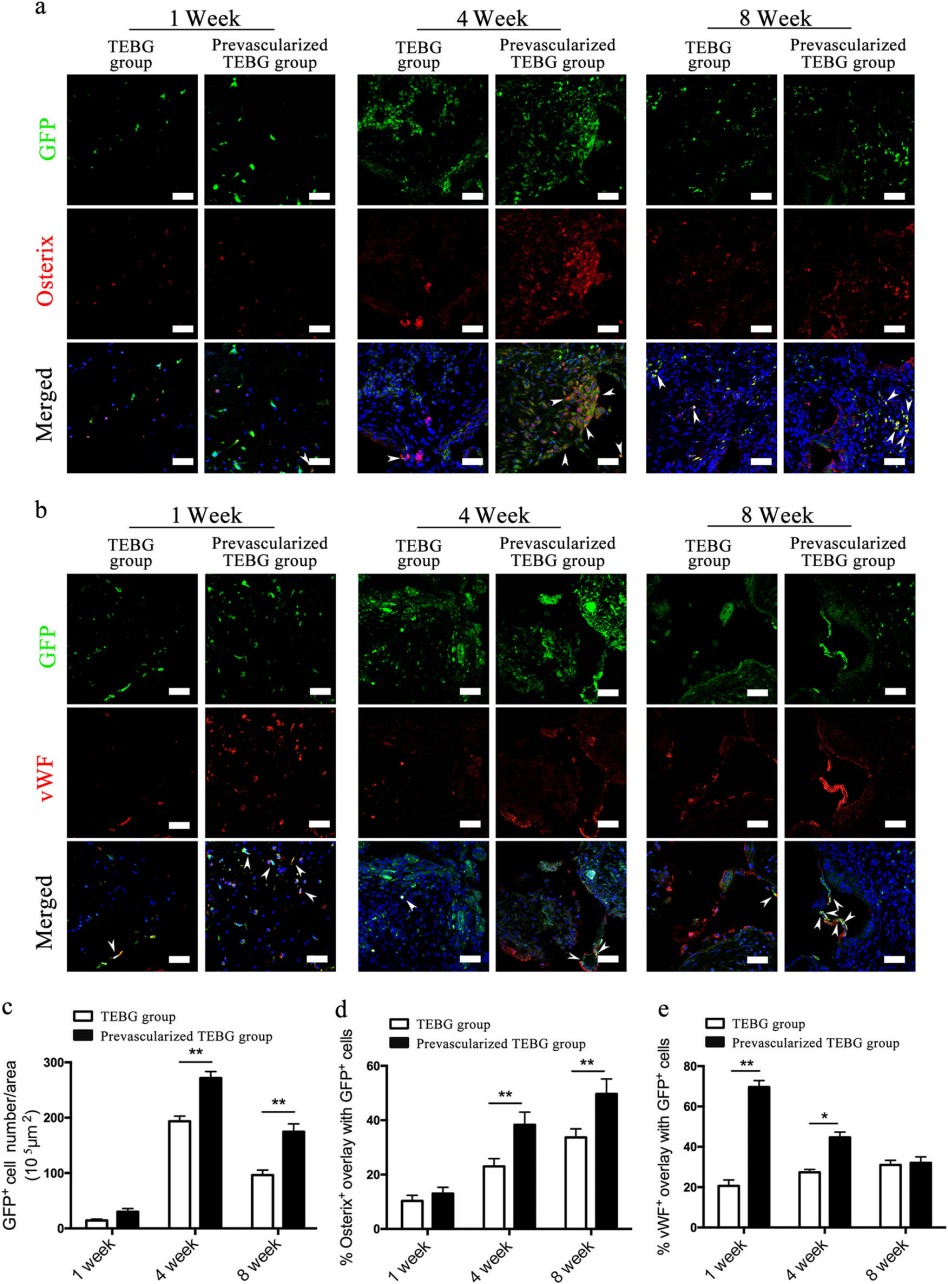
Prevascularization of TEBGs promoted proliferation and differentiation of seed cells. Image was the center region of transection. Double-immunofluorescence images of BMSCs (green) and Osterix (red; **a**) or vWF (red; **b**) from TEBG sections, scale bar = 50 μm. White arrows represent double-positive staining cells. Hoechst33342 stained the nuclei blue. **a**, **c** Seeding cells continued to survive after implantation, and the number of seeding cells reached the peak at 4 weeks. **c** The number of GFP^+^ cells per area (10^5^ μm^2^), *n* = 10. **d** Percentage of Osterix^+^ cells overlapped with GFP^+^ cells, *n* = 10. **e** Percentage of vWF^+^ cells overlapped with GFP^+^ cells, *n* = 10. Data are represented as the mean ± s.e.m. *P < 0.05, **P < 0.01 as determined by Student’s *t*-tests

The fate of seeded cells in vivo was first observed by implanting GFP^+^ BMSCs-TEBG into the femur defects of WT rats. By tracing these seeded cells by detecting GFP fluorescence, we found that they continued to survive for at least 8 weeks after implantation (Fig. 3a, b). Prevascularization significantly increased the number of GFP^+^ cells 4 weeks after implantation compared with the number in the TEBG group (Fig. 3c).

The osteogenic differentiation fates of GFP^+^ seeding cells was first detected by immunofluorescence of Osterix. GFP^+^ seeding cells in TEBGs underwent osteoblast differentiation in vivo but at a low ratio at 1 week after implantation. This ratio was elevated 4 weeks after implantation and reached a peak at 8 weeks (Fig. 3d). Prevascularization of TEBGs significantly increased the osteoblast differentiation of GFP^+^ seeding cells from 4 weeks after implantation compared with that in the control TEBG group.

Unlike osteoblast differentiation, the endothelial differentiation of GFP^+^ seeding cells displayed a different pattern. Compared with the in vivo osteoblast differentiation of seeding cells, the endothelial differentiation occurred much earlier. 1 week after implantation, a greater number of GFP^+^ seeded cells in the prevascularized TEBG group underwent endothelial differentiation, as evidenced by immunofluorescence staining for vWF (Fig. 3b). However, the percentage of vWF^+^ seeded cells was substantially reduced in both the prevascularized group and the control group at 8 weeks, whereas some vWF^+^ vessel-like structures were observed in the prevascularized group (Fig. 3b–e). These results were the first to show that BMSCs differentiated into endothelial cells in TEBG-mediated bone regeneration. More interestingly, prevascularization increased the endothelial differentiation of seeded BMSCs.

The in vivo cell fate of host cells was also observed by implanting WT BMSCs-TEBGs into GFP^+^ rat femur defects and tracing GFP florescence of host cells.

**Fig. 4:**
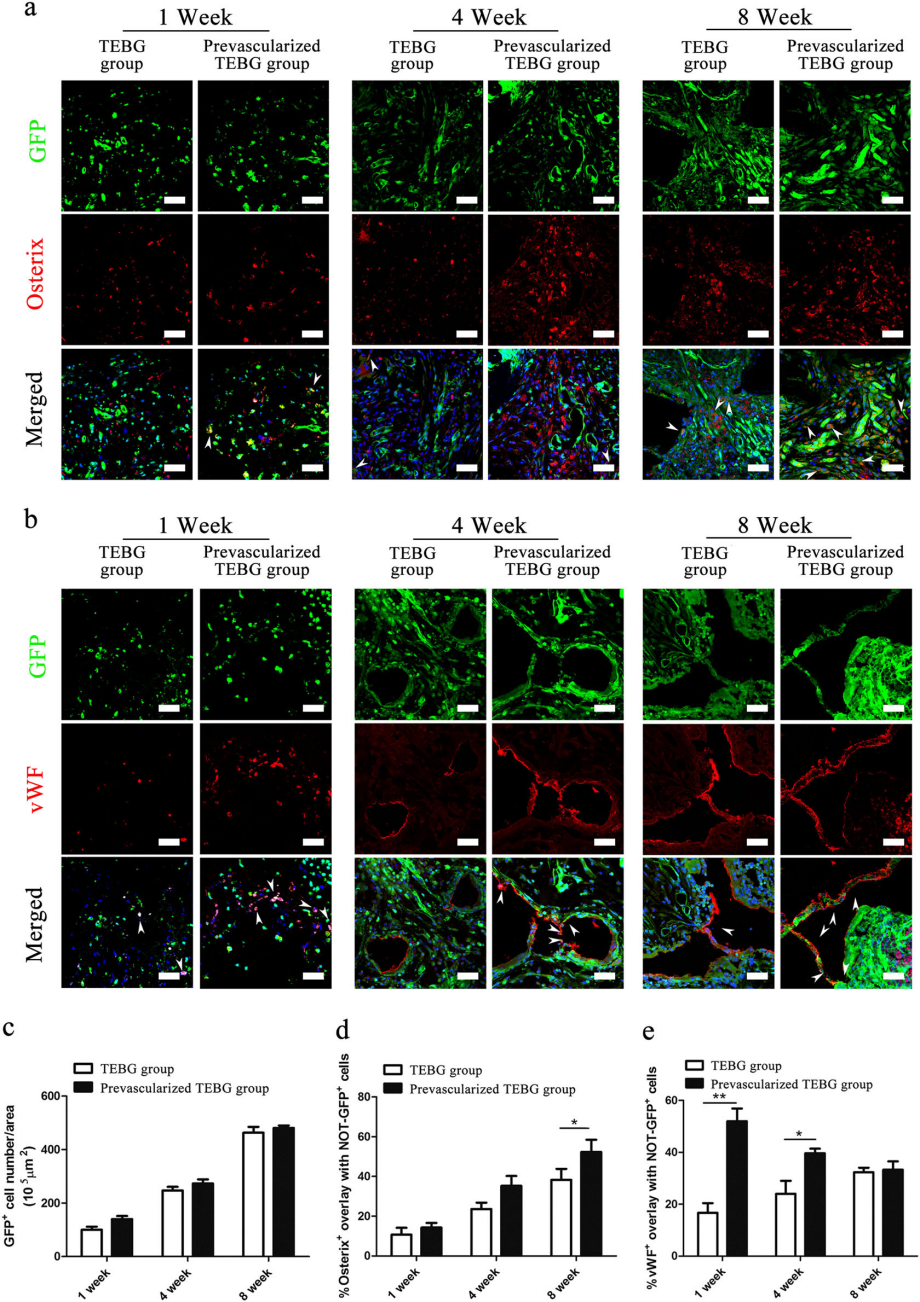
Prevascularization of TEBGs promoted osteogenic and endothelial differentiation of host cells. Double-immunofluorescence images of BMSCs (green) and Osterix (red; **a**) or vWF (red; **b**) from TEBG sections, scale bar = 50 μm. White arrows represent double-positive staining cells. Hoechst33342 stained the nuclei blue. **a**, **c** Host cells migrated into the TEBG pores. **c** The number of GFP^+^ cells per area (10^5^μm^2^), *n* = 10. **d** Percentage of Osterix^+^ cells overlapped with NOT-GFP^+^ cells, *n* = 10. **e** Percentage of vWF^+^ cells overlapped with NOT-GFP^+^ cells, *n* = 10. Data are represented as the mean ± s.e.m. *P < 0.05 as determined by Student’s t-tests

At 1 week after implantation, many GFP^+^ host cells migrated into TEBG pores, and the number of host cells noticeably increased over time (Fig. 4a, b). The possible reasons for the increasing number of host cells might include both continuing migration and self-proliferation. However, a significant difference in the number of GFP^+^ host cells was not observed between the prevascularized group and the control group (Fig. 4c). Consistent with the results from the GFP^+^ BMSCs-TEBG model, prevascularization also increased osteogenic differentiation at 4 weeks and endothelial differentiation at 1 week after implantation (Fig. 4d, e).

### Co-culture with RAOECs promoted the endothelial differentiation of BMSCs

**Fig. 5:**
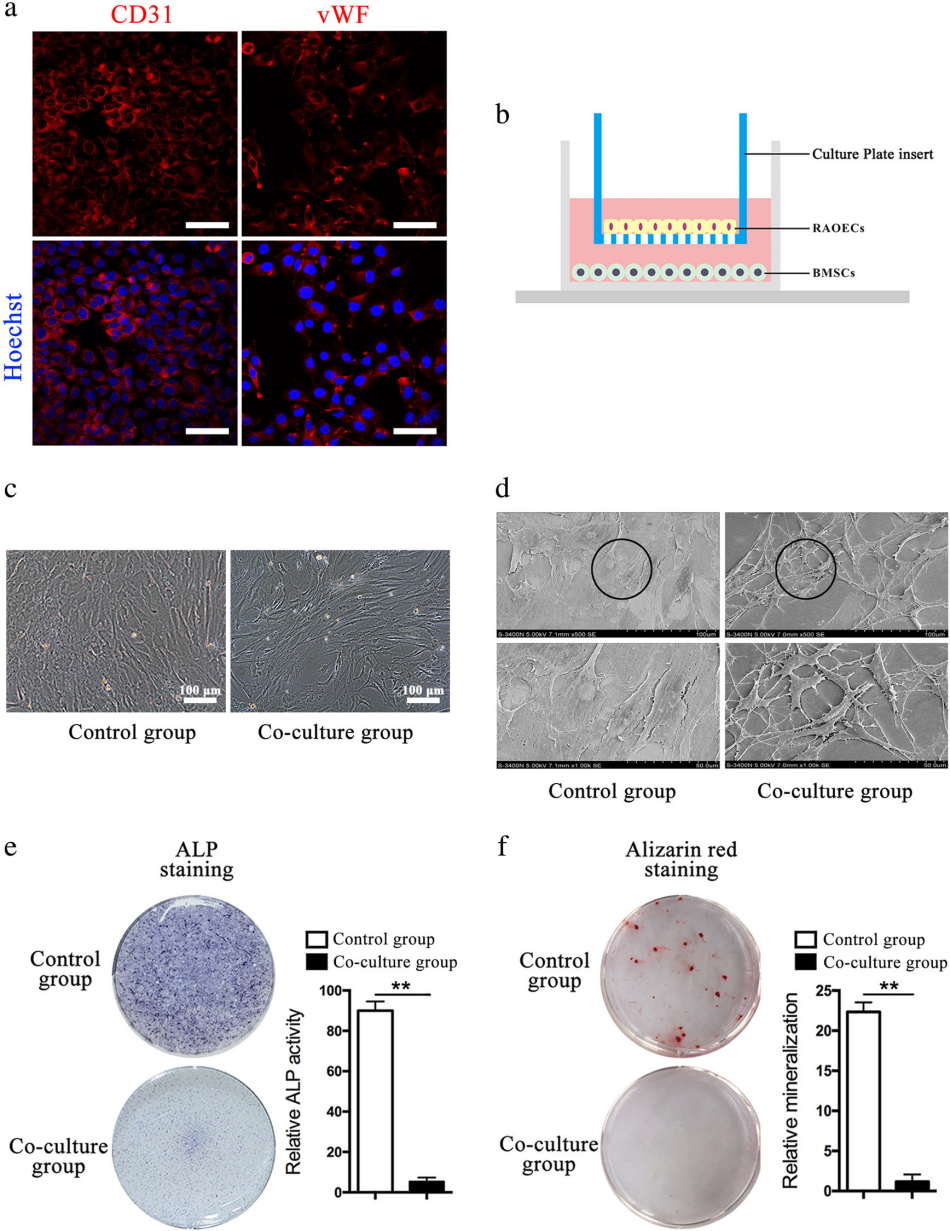
BMSCs were morphologically altered after being co-cultured with RAOECs. Immunofluorescence images of CD31 (red: **a** left panel) and vWF (red; **a** right panel); Hoechst33342 stained the nuclei blue, scale bar = 50 μm. **b** Schematic drawing of co-culture system. Representative images of BMSCs under an optical microscope (100 × ) (**c**) and an SEM (×500, ×1000) (**d**). BMSCs in the control group and the co-culture group were stained by ALP after 14 days of culture (**e**, left panel). BMSCs in the control group and the co-culture group were stained by Alizarin Red S after 21 days of culture (**f**, left panel). *n* = 3. Data are represented as the mean ± s.e.m. **P < 0.01 as determined by Student’s *t*-tests

Rat aortic endothelial cells (RAOECs) exhibited positive staining for both CD31 (cluster of differentiation 31) and vWF (Fig. 5a). We established a co-culture system using transwell chambers to observe how the RAOECs influence BMSCs (Fig. 5b). After 7 days of co-culture, the BMSCs displayed a vimineous morphology, which was further supported by SEM observations (Fig. 5c, d). The obvious morphology of BMSCs in co-culture indicated that the cell fate of BMSC differentiation is altered with the influence of RAOECs.

**Fig. 6:**
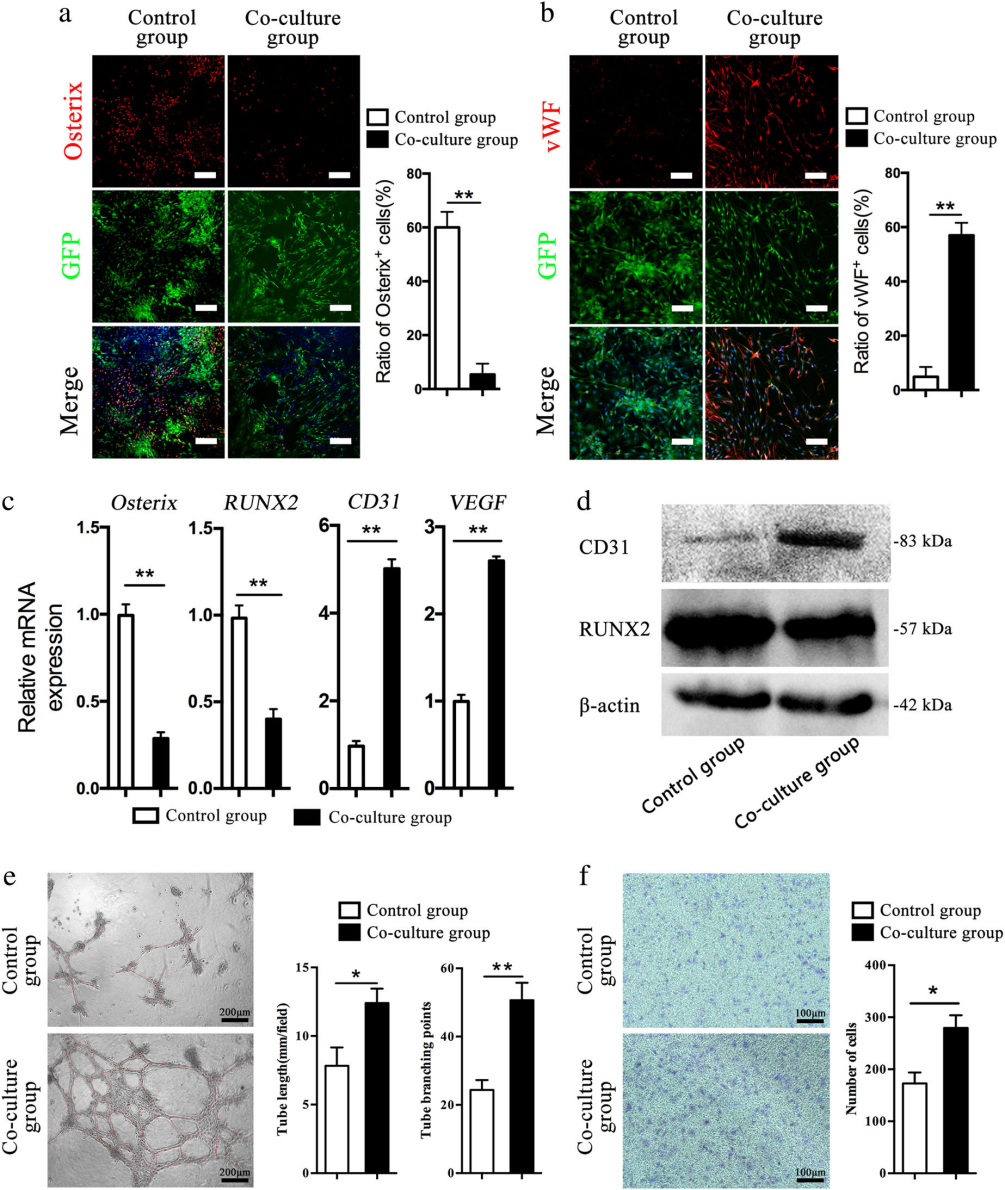
BMSCs differentiated into endothelial-like cells in the co-culture system. Immunofluorescence staining of BMSCs using antibodies against Osterix (**a**, shown in red, scale bar = 200 μm) and vWF (**b**, shown in red, scale bar = 100 μm). Ratio of Osterix^+^ cells (%), *n* = 3 (**a**, right panel). Ratio of vWF + cells (%), *n* = 3 (**b**, right panel). **c** Relative mRNA expression of Osterix, RUNX2, CD31 and VEGF, *n* = 3. Data are represented as the mean ± s.e.m. **P < 0.01 as determined by Student’s *t*-tests. **d** WB analysis of CD31, RUNX2 and β-actin expression in BMSCs between the control and co-culture groups. **e** Representative images of the tube-like structures for the two groups. Images were taken at ×100 magnification. Tube formation assay analysis was carried out using the Wimasis platform, plotted as the number of tube length and branching points. Results are expressed relative to control. *n* = 3. Data are represented as the mean ± s.e.m. *P < 0.05; **P < 0.01 as determined by Student’s *t*-tests. **f** Representative images of the Crystal Violet staining for the two groups. Cells couting was using Image J. Results are expressed relative to control. *n* = 3. Data are represented as the mean ± s.e.m. *P < 0.05 as determined by Student’s t-tests

The osteogenic differentiation of BMSCs was significantly reduced upon co-culture with RAOECs compared with BMSCs cultured alone (Fig. 5e). Compared with the control, there were few calcium nodules (Fig. 5f). The osteogenic differentiation of BMSCs in the co-culture group was significantly reduced compared with the control culture. We further tested osteogenic differentiation by performing immunofluorescence staining with an anti-Osterix antibody (Fig. 6a) and found that BMSCs in the co-culture group showed a lower level of Osterix expression. Thus, these series of tests demonstrated the RAOECs inhibited BMSC differentiation into osteoblasts, even under osteogenic induction.

However, when two groups of BMSCs were cultured in regular medium for 14 days, BMSCs were positively detected by immunofluorescence staining with an anti-vWF antibody (Fig. 6b). The expression of the osteogenic genes, Osterix and runt-related transcription factor 2 (Runx2), was markedly downregulated in co-cultured BMSCs compared with their expression in control cultures. Meanwhile, the expression of the angiogenic genes CD31 and vascular endothelial growth factor (VEGF) was markedly upregulated in co-cultured BMSCs compared with BMSCs cultured alone (Fig. 6c).

In addition, the levels of the Runx2 protein were decreased in the co-culture group, whereas CD31 levels were obviously increased in the co-culture BMSCs (Fig. 6d). All quantitative real-time polymerase chain reaction (qRT-PCR) and Western blotting (WB) results indicated that RAOECs promoted BMSCs differentiation into endothelial cells with less osteogenic differentiation.

BMSCs cultured with RAOECs had a greater number of capillary-like tube structures than those in the negative control group. A further analysis of capillary-like tube formation by BMSCs in the co-culture and control groups revealed significantly longer tubes and more branch points in the presence of RAOECs, and this effect likely depended on exosomes from RAOECs (Fig. 6e). Next, we assessed the effect of RAOECs on BMSC migration by performing a cell migration assay. There were more stained cells in the co-culture group than in the control group (Fig. 6f). The ability of BMSCs to form tubes and migrate increased after culture with RAOECs. The results indicate that BMSCs in the co-culture group were prompted to differentiate into endothelial cells and adopt the functions of endothelial cells.

### RAOECs inhibited RhoA/ROCK signaling in BMSCs

**Fig. 7:**
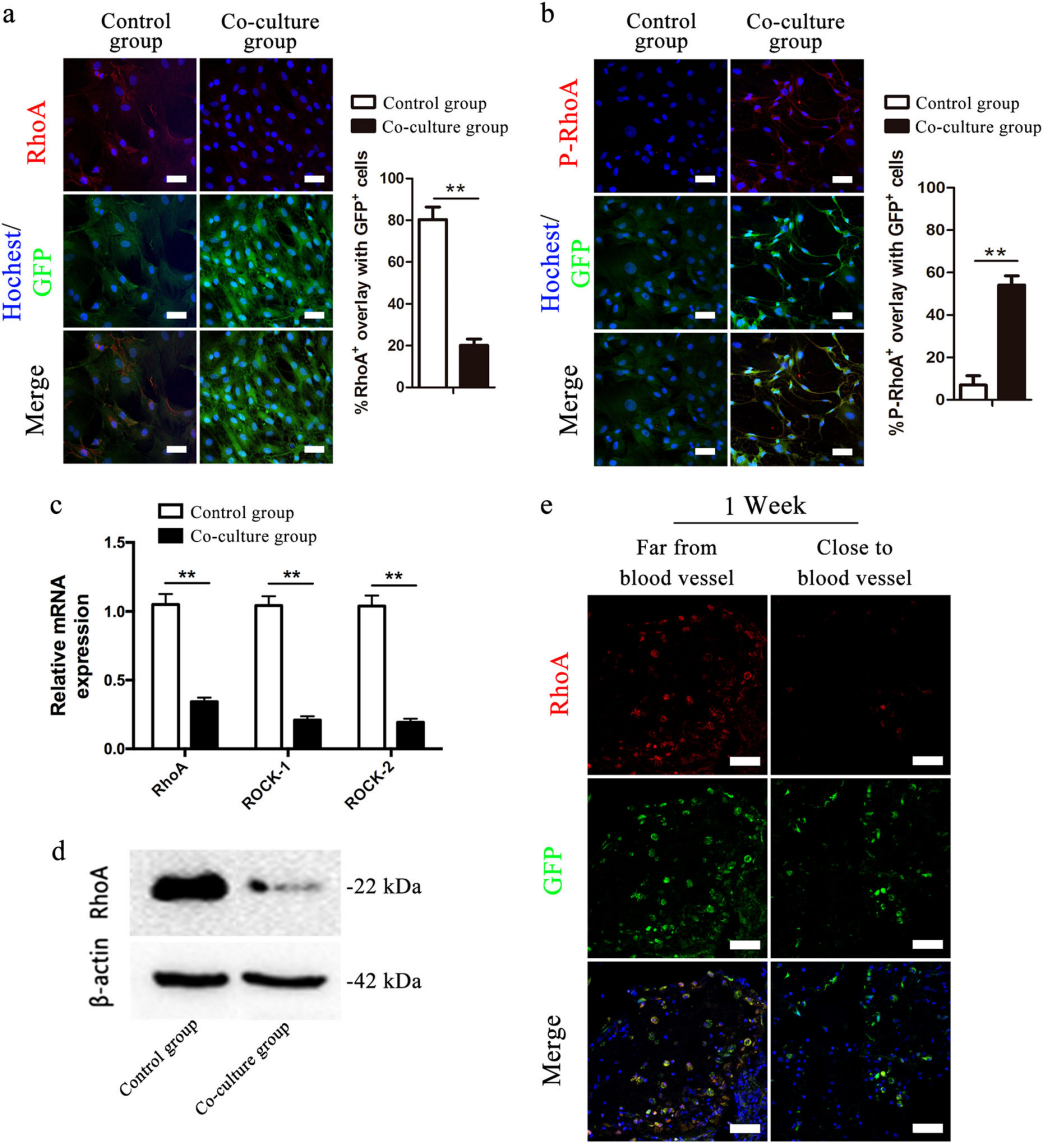
RAOECs inhibited the RhoA/ROCK signaling pathway in BMSCs. Double-immunofluorescence images of BMSCs (green) with RhoA (red; **a**) or p-RhoA (red; **b**); Hoechst33342 stained the nuclei blue, scale bar = 50 μm. Percentage of RhoA^+^ cells overlapped with GFP^+^ cells (**a**, right panel). Percentage of p-RhoA^+^ cells overlapped with GFP^+^ cells (**b**, right panel). (**c**) qRT-PCR analysis of RhoA, ROCK-1 and ROCK-2 in BMSCs in the control group and the co-culture group, *n* = 3. Data are represented as the mean ± s.e.m. **P < 0.01 as determined by Student’s t-tests. **d** WB analysis of RhoA expression in BMSCs. **e** Immunofluorescence images of BMSCs (green) and RhoA (red) in TEBG sections, scale bar = 50 μm

The cytoplasmic staining for the total RhoA protein was obviously reduced in BMSCs co-cultured with RAOECs compared with the staining in control BMSCs (Fig. 7a). In contrast, the level of phosphorylated RhoA (p-RhoA, a marker of the inactive form of RhoA)^32^ was significantly increased in BMSCs co-cultured with RAOECs (Fig. 7b), indicating that RhoA signaling was inhibited when BMSCs were co-cultured with RAOECs. Together with the downregulated expression of RhoA, the decreased expression of the RhoA, ROCK-1, and ROCK-2 transcripts (Fig. 7c, d) showed that the RhoA/ROCK pathway was significantly inhibited in BMSCs co-cultured with RAOECs.

In vivo, scaffold pores located close to the vascular bundle exhibited fewer RhoA-positive cells than the pores located far away from the vascular bundle (Fig. 7e). These interesting results were consistent with the in vitro data, suggesting that vascularization inhibited the RhoA/ROCK signaling pathway.

### FGF2 secreted by RAOECs determined the endothelial differentiation of BMSCs by modulating the RhoA/ROCK signaling pathway

**Fig. 8:**
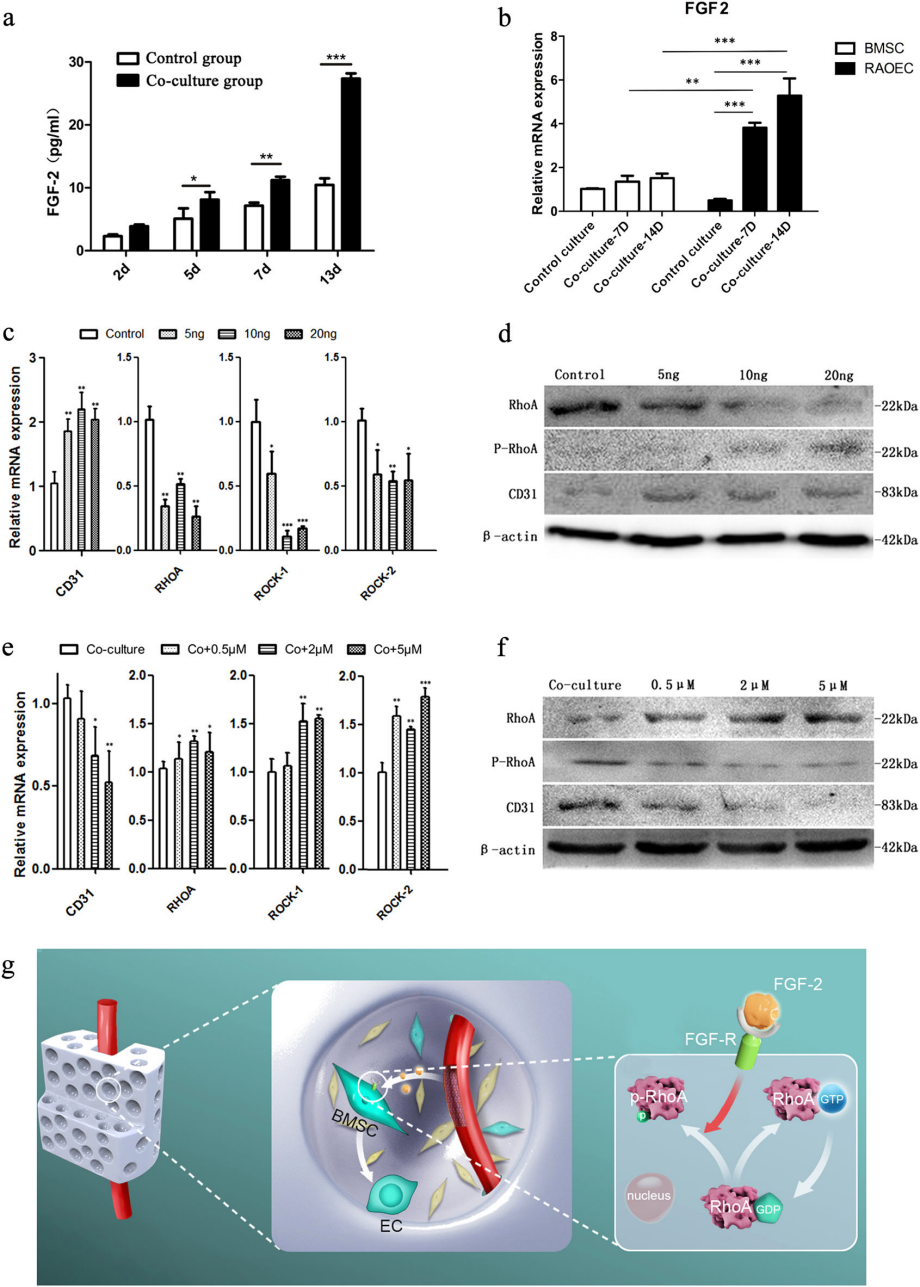
RAOECs regulate BMSC differentiation into endothelial cells via the FGF2-RhoA/ROCK signaling pathway. **a** Level of FGF2 in the control group and the co-culture group, *n* = 3. Data are represented as the mean ± s.e.m. *P < 0.05; **P < 0.01; ***P < 0.001as determined by Student’s *t*-tests. **b** Relative FGF2 mRNA expression in the control group and the co-culture group, *n* = 3. Data are represented as the mean ± s.e.m. **P < 0.01; ***P < 0.001 as determined by Student’s t-tests. **c**, **e** qRT-PCR analysis of RhoA, ROCK-1, ROCK-2 and CD31 in BMSCs from groups with different treatments, *n* = 3. Data are represented as the mean ± s.e.m. *P < 0.05; **P < 0.01; ***P < 0.001 as determined by Student’s t-tests. **d**, **f** WB analysis of RhoA, ROCK-1, ROCK-2 and CD31 in BMSCs from groups with different treatments. **g** FGF2, secreted by endothelial cells, binds with FGFRs on BMSC membranes and promotes GDP-RhoA translation into inactive p-RhoA but not active GTP-RhoA

We detected FGF2 levels in the co-culture medium by ELISA assay. The data are summarized in a chart (Fig. 8a) showing distinctly higher FGF2 levels in the co-culture medium compared with the control medium, and the difference between the two groups increased over time.

The qRT-PCR data (Fig. 8b) showing a higher expression level of the FGF2 gene in the RAOECs than in the BMSCs, and the difference between the two groups increased over time in co-culture. The qRT-PCR result was consistent with the ELISA data, confirming that FGF2 was secreted by RAOECs and not BMSCs.

After 7 days of culture with FGF2, BMSCs were collected to extract RNA and protein. The qRT-PCR results indicated the FGF2 inhibited the RhoA/ROCK pathway by reducing the expression of RhoA, ROCK-1 and ROCK-2. Consistent with these findings, the addition of FGF2 promoted the expression of CD31 (Fig. 8c). Similarly, the WB results showed that RhoA expression was downregulated with the increased FGF2 dosage. In addition, elevated levels of p-RhoA and CD31 were detected in the 20 ng FGF2 group (Fig. 8d). This suggested that FGF2 was a stronger RhoA inhibitor.

Furthermore, we added different doses of FGF2 inhibitor to the co-culture system to observe whether the endothelial differentiation of BMSCs induced by FGF2 could be ablated. According to the qRT-PCR results, CD31 was downregulated and the levels of RhoA/ROCK signaling-related genes were upregulated by the FGF2 inhibitor (Fig. 8e). Moreover, the level of the RhoA protein was increased and the levels of the p-RhoA and CD31 proteins were simultaneously reduced (Fig. 8f), further confirming that the effect of vascularization was ablated by the FGF2/FGFR inhibitor. In summary, these in vitro results clarified that FGF2-mediated RhoA/ROCK inhibition was a key modulator of endothelial differentiation in BMSCs.

## Discussion

Since 1990s, great progress has been made in tissue engineering, such as in seeding cells, scaffold development, animal models and clinical trials. However, the in vivo fate of seeding cells and the cell components within regenerated new bone remained unclear. A key obstacle researchers face is the in vivo labeling of seeding cells and host cells. By labeling seeding cells or host cells with GFP, the expansion and survival of seeding cells and host cells in TEBGs were clearly displayed within 8 weeks after implantation.

This alternative in vivo tracing model was also used to understand the cell fate change of seeding BMSCs and the stimulatory effect of prevascularization on tissue-engineered bone regeneration. These studies strongly suggested a new mechanism underlying the effect of prevascularization on bone regeneration. Our results showed that the FGF2-RhoA/ROCK signaling pathway was a key determinant for the lineage fate of BMSCs in the initial vascularization of TEBGs. After the translocation of vessel bundles in the implantation operation, the endothelial cells in prevascularized TEBGs secreted FGF2 to inhibit the RhoA/ROCK signaling pathway in surrounding BMSCs (Fig. 8g). The inhibition of RhoA/ROCK converted BMSC differentiation toward endothelial lineage cells in the initial stage. Four weeks following implantation and after, the endothelial cells from BMSCs formed vessel-like tubular structures, which may transport more oxygen and nutrients into the middle part of TEBGs and promote bone regeneration.

The aim of our research was to study the effects of vascularization on the survival and differentiation of BMSCs in vivo and in vitro. The transplanted vascular bundle enhanced the differentiation of seeded BMSCs into endothelial cells in the early implantation stage. Under some conditions, such as inflammatory conditions, the vasculature continuously leaks the solute and small cytokines but restricts the extravasation of larger molecules and cells^33^. Vascular permeability increases, and the vasculature finally becomes a semipermeable membrane-like structure that segregates ECs and BMSCs. As a continuation of and complement to the in vivo experiment, the in vitro experiment also needed a semipermeable membrane to segregate BMSCs from ECs. Therefore, we chose a co-culture system that prevented direct contact between these cells.

In our non-direct contact co-culture system, significantly higher FGF2 levels were detected in the co-culture medium compared with the medium from BMSCs cultured alone, as determined by an ELISA. Accordingly, we hypothesized that RAOECs secreted FGF2 to regulate BMSC differentiation. FGF2 is considered a differentiation inducer and regulatory factor in the stem cell research that exerts diverse effects on cell proliferation and differentiation. The discrepancy might be attributed to differences in the culture systems, culture conditions and cell status.

FGF2 was suggested to promote osteogenesis as synergistic factor. The combination of FGF2 with the active form of vitamin D induced osteodifferentiation. Thus, the FGF2 treatment might potentiate the effects of 1,25-(OH)_2_D_3_ on ALP activity and mineralization in osteoblasts by upregulating the transactivating activity of Runx2^34^. In the presence of a constant amount of BMP-2, some studies confirm that low doses of FGF-2 increase ectopic bone formation in vivo by upregulating Runx2 expression and increasing ALP activity. BMPs belong to the transforming growth factor superfamily and play crucial roles in osteogenesis and bone metabolism. Among these growth factors, BMP-2 has a very strong osteoinductive activity^35,36^. In summary, FGF-2 may cooperate with other cytokines to directly promote osteogenesis; these effects cannot be separated from the osteogenic activity of these cytokines.

In addition, FGF2 clearly promotes bone formation by increasing angiogenesis^37^. FGF2 stimulates the proliferative potential of fibroblasts and angiogenesis to accelerate the closure of defects during the wound healing process^38^. The formation of new blood vessels is of particular importance in the regeneration of defect sites; thus, FGF2, an angiogenesis regulator, and other cytokines play key roles in the osteogenesis and bone formation processes^39^. In our in vivo experiment (Fig. 3a, b), the cells (BMSCs) seeded in TEBGs were differentiated into vWF^+^ cells within 1 week after implantation. However, a large number of BMSCs differentiated into Osterix^+^ cells in the later stage. We were not able to exclude the possibility that FGF2 promotes angiogenesis in the early stage, and following the formation of new blood vessels, regeneration, osteogenesis and bone formation are also promoted in the later stage.

Finally, the role of FGF2 in osteoblastic differentiation depends on the cell type and maturation stage and the FGF2 concentration^40^. According to some studies, excess levels of high-molecular weight (HMW) FGF2 isoforms negatively regulate BMSC mineralization in vitro^41^. Based on the data presented in the study by Emmanuel B, FGF2 modulates the BMP pathway in HMSCs by downregulating BMP/BMPR expression, thereby inhibiting the osteoblastic differentiation of HMSCs. This effect is mediated by the ERK and JNK MAPKs pathways. FGF2 alone induces HMSC proliferation and inhibits ALP activity and HMSC mineralization. These data provide an additional mechanism of crosstalk between growth factors and FGF2^42^.

In conclusion, FGF2 exerts diverse effects on different systems, not only promoting osteogenic differentiation and angiogenesis but also inhibiting osteogenic differentiation under some special conditions.

In summary, for the first time, our study identified the FGF2-RhoA/ROCK axis as a molecular switch of BMSC fate during bone defect repair. The results presented a creative explanation for prevascularization to accelerate bone regeneration. In addition, this molecular mechanism can be used to establish fast-acting angiogenesis materials in future clinical applications. Since BMSCs were shown to differentiate into endothelial cells in the early stage of regeneration, it is unnecessary to combine TEBGs with exogenous endothelial cells or vessels. This finding will reduce the complexity and time of TEBG implantation.

## Materials and methods

### Animals and ethics statement

WT Sprague-Dawley (SD) rats were obtained from the Experimental Animal Center of the Fourth Military Medical University. GFP^+^ SD rats were purchased from Xing Ming Biomedical Technology Co., Ltd. (Shanghai, China). All rats were maintained in the animal facility of the Experimental Animal Center of the Fourth Military Medical University. The experimental protocol was reviewed and approved by the Ethics Committee for Animal Research of the Fourth Military Medical University (approval number 20150405).

### BMSC isolation, culture, and identification

The WT BMSCs and GFP^+^ BMSCs were isolated from the femur bone marrow of 2-week-old WT and GFP^+^ rats, respectively. First, the immature rats were euthanized by pentobarbital overdose via an intraperitoneal injection. Second, the femurs were separated from muscle. After washes with phosphate-buffered saline (PBS), the metaphysis was removed. Finally, the marrow was rinsed and placed in a petri dish with complete medium. The complete medium was alpha-modified minimum essential medium (α-MEM, HyClone, Logan, UT, USA) supplemented with 10% fetal calf serum (FCS), 5% L-glutamine (both from Gibco, New York, NY, USA), and 1% streptomycin/penicillin antibiotics (Solarbio, Beijing, China). After 24 h, the non-adherent cells were gently removed by three rinses with PBS (HyClone, USA). Every 72 h, the culture medium was exchanged to remove the dead and non-adherent cells. When the cells were 70–80% confluent, adherent cells were trypsinized, harvested, and subcultured into new dishes at a density of 1.0 × 10^5^ cells/ml. After 3 weeks of culture and 3 passages, BMSCs were seeded into the scaffold grafts.

BMSCs (5.0 × 10^5^ cells, passage 3) were collected in PBS and sequentially incubated with CD31^−^PE (555027, BD, San Jose, CA, USA), CD11b/c-FITC (201805, Biolegend, USA), CD45-PE-Cy7 (202214, Biolegend, San Diego, CA, USA) and CD90-PerCP (202512, Biolegend, USA) for 30 min at 37 °C in the dark. Data were acquired using a FACSCanto II flow cytometer (BD, USA) and analyzed with FACSDiva software version 6.1.3 (BD, USA).

The differentiation potential of rat BMSCs was further verified by induction with osteogenic, adipogenic and chondrogenic media (all from Cyagen, Santa Clara, CA, USA) according to the manufacturer’s protocol. For osteogenic differentiation, after 21 days of induction in osteogenic medium, cells were fixed with a 4% paraformaldehyde (PFA) solution (HEART, Xi’an, China) for 10 min and stained with Alizarin Red S (Beyotime, Shanghai, China) for 3 min. For adipogenic differentiation, cells were fixed with 4% PFA for 10 min and stained with Oil Red O (Heart, China) for 30 min after 27 days of induction. For chondrogenic differentiation, 1.0 × 10^6^ cells were collected in 15-ml polypropylene culture tubes, centrifuged at 150 *×* *g* for 5 min to form cell pellets, and then cells were resuspended in complete chondrogenic medium to a concentration of 5.0 × 10^5^ cells/ml. Aliquots (0.5 ml, 2.5 × 10^5^ cells) of the cell suspension were transferred into 15-ml polypropylene culture tubes and centrifuged at 150 *×* *g* for 5 min at room temperature. The caps of the tubes were loosened one half turn to allow gas exchange, and the tubes were incubated at 37 °C in a humidified atmosphere of 5% CO_2_. After 28 days, the cell pellets were prepared into frozen 7-μm-thick sections and stained with Alcian Blue (Heart, China) for 30 min. All images were obtained using an inverted microscope (Axio Observer A1, Carl Zeiss, Oberkochen, Germany).

### Preparation of TEBGs

Porous β-tricalcium phosphate scaffolds (β-TCP, 4 × 5 mm^2^, pore diameter 400 µm, 70% porosity) with a 1^−^mm-deep lateral groove were adapted to a rat (Bio-lu Biomaterials, Shanghai, China). BMSCs were used as seed cells. In addition, passage 3 cells were collected and resuspended at a concentration of 5 × 10^6^ cells/ml. Forty microliters of the suspended cells were carefully seeded onto each scaffold in 24-well plates. Then, the cell-scaffold complexes were placed in an incubator for 2 h to promote the tight adhesion of cells to the scaffold. Afterwards, 1 ml of complete medium was added to each well, and the complexes were incubated for 7 days before implantation. Because we used two types of BMSCs (WT and GFP^+^), two types of tissue-engineered bone grafts (GFP^+^ BMSCs-TEBG and WT BMSCs-TEBG) were successfully constructed.

### Large bone (rat femur) defect model

According to a previous study^31^, a 5-mm bone defect in the rat femur is regarded as a critical size defect that does not undergo self-repair. Rats (female, 10 weeks old, 270 ± 10 g) were fasted overnight before the surgical procedure. Rats were anesthetized via an intraperitoneal injection of 1% w/v pentobarbital sodium salt (40 mg/kg, Merck, Darmstadt, Germany). After being shaved and sterilized, the first incision on lateral femoral was made to expose the middle femoral shaft, and a large bone defect (5 mm in length) was created in the left femur and fixed by internal fixation. A second longitudinal incision was made in the groin to expose the femoral blood vessel. Segments of the femoral vein and artery were isolated and implanted onto the side groove of the TEBG through the muscle (prevascularized TEBG group). The rats that were implanted with the TEBG without the blood vessels were designated the TEBG group^43^.

Sixty WT SD rats were randomly divided into two groups of 30 rats each: the prevascularized TEBG group and the TEBG group. The WT SD rats were matched with GFP^+^ BMSCs-TEBGs to locate the implanted GFP^+^ BMSCs and investigate BMSC differentiation by detecting GFP expression.

Sixty GFP^+^ SD rats were also randomly divided into two groups of 30 rats each: the prevascularized TEBG group and TEBG group. The GFP^+^ SD rats were matched with WT BMSCs-TEBGs to explore differentiation of the host cells by detecting GFP expression.

All animals undergoing surgery were kept warm with an electric blanket during the operation and for 3 h afterwards.

### X-ray examination and Micro-CT analysis

Ten rats from each group were humanely euthanized at 1, 4, or 8 weeks after surgery for subsequent examinations. The femurs were carefully removed after rats were perfused with a 4% PFA solution. Plain X-ray images were obtained at 35 kV and 1.5 mA for 3 s using an X-ray machine (Carestream DRX Ascend, Carestream Health, Toronto, ON, Canada). Microcomputed tomograms (micro-CTs) of harvested femora were acquired with a micro-CT system (GE Healthcare, Fairfield, CT, USA). The X-ray source voltage was set to 80 kV and anode current to 80 mA; the scanning angular rotation was 360° and the angular increment was 0.50°. Microview v2.1.2 software was used for the analysis. The projections were reconstructed, and a 5 × 12.56 mm^2^ cylindrical region was selected as the region of interest (ROI). Thresholds were applied to differentiate between new bone and residual scaffold material in the ROI using previously reported methods^44,45^. A CT value ranging from 900 Hu to 2000 Hu was regarded as newly formed bone. The rate of new bone formation was evaluated by the bone volume fraction, BV/TV, which was calculated as the volume of the new bone formation divided by the total volume of the ROI. A CT value greater than 5000 Hu was regarded as an unmodified scaffold, according to manufacturer’s data specification sheets. The scaffold degradation rate (RSV/SV) was calculated as the resident scaffold volume (RSV) divided by the scaffold total volume (SV) before implantation^46^.

### Histological analysis

After radiological monitoring, samples were fixed with a 4% paraformaldehyde solution for 3 days and decalcified with 10% ethylenediaminetetraacetic acid (EDTA, HEART, China) for 4 weeks on a shaking table at room temperature. The decalcification procedure was complete when the bone was soft enough to be easily stabbed with a needle. Afterwards, samples were dehydrated in a mixture of 30% sucrose and 10% gum arabic for three days at 4 °C^47^. Frozen sections (10 μm) of the decalcified specimens were serially cut parallel and perpendicular to the long axis of the femur using a freezing microtome (Leica, Wetzlar, Germany). All sections were preserved at −20 °C. In addition, longitudinal sections were stained with hematoxylin and eosin (H&E). The sections were observed using an inverted microscope (BX53, Olympus, Tokyo, Japan).

### Immunofluorescence staining

Frozen bone sections from each group were randomly divided into two groups. After rewarming at 37 °C for 30 min, the sections were washed with PBS three times, then permeabilized with 1% Triton X-100 (Sigma Aldrich, St. Louis, MO, USA) for 10 min at room temperature and rinsed well with PBS three times. All sections were incubated with blocking buffer (1% donkey serum, Solarbio, China) for 30 min to block nonspecific antibody binding sites. After this step, the sections were incubated with one of the primary antibody solutions, anti-vWF (ab6994, 1:200, Abcam, Cambridge, MA, USA) or anti-Osterix (ab22552, 1:200, Abcam, USA), at 4 °C overnight, followed by an incubation with a secondary antibody solution containing donkey anti-rabbit IgG H&L (Alexa Fluor^®^ 594) (ab150076, 1:1000, Abcam, USA) for 1 h. Nuclei were stained with Hoechst33342 (Sigma Aldrich, USA) for 5 min. Sections were mounted with Enhanced Antifade Mounting Medium (Leagene, Beijing, China). Images of the center field of view in ten independent samples were captured using a laser scanning confocal microscope (A1R, Nikon, Tokyo, Japan). Cell counts were quantified using ImageJ software^48^.

### Identification of rat aortic endothelial cells

RAOECs, which were purchased from Cell Applications (San Diego, CA, USA), were cultured in the same medium as BMSCs. All cells were cultured at 37 °C in a 5% CO_2_ atmosphere. RAOECs were identified by immunofluorescence staining. Cells were fixed with 4% PFA for 10 min and then incubated with 1% donkey serum for 30 min to block nonspecific protein-protein interactions. Then, cells were incubated with anti-CD31 (ab64543, 1:200, Abcam, USA) or anti-vWF (1:200) overnight at 4 °C. The secondary antibody (red), donkey anti-mouse IgG H&L (Alexa Fluor® 594, ab150108, 1:1000, Abcam, USA) or donkey anti-rabbit IgG H&L (Alexa Fluor® 594, 1:1000, Abcam, USA), was incubated with the cells for 1 h. A 1:1000 dilution of Hoechst33342 was incubated with cells for 5 min to stain the cell nuclei (blue).

### Co-culture system

RAOECs and BMSCs were used to establish a co-culture system. RAOECs were cultured in transwell chambers, and BMSCs were cultures in the bottoms of the 6-well plates. The two types of cells were separated with a culture plate insert (PIHP03050, Millipore Millicell, MA, USA). Both cell lines were inoculated at a density of 2000 cells per well in the co-culture group. The control group was BMSCs alone. After 7 days of culture, the BMSCs in the control group and the co-culture group were observed using an inverted microscope (AXIO Observer. A1, Carl Zeiss, Germany) and a scanning electron microscope (SEM, Hitachi, Tokyo, Japan). The samples were fixed in 4 °C 3% glutaraldehyde solution for 2 h. After a series of professional treatments, the samples were examined using an energy dispersive spectrometer (EDS) integrated within the SEM. Integrated mapping software was used to analyze the element distribution.

### Differentiation of BMSCs in the co-culture system

The control group and the co-culture group were cultured in osteogenic medium to examine the osteogenic differentiation of BMSCs in the co-culture system. After 14 days of culture, the BMSCs in the two groups were fixed with 4% PFA for 10 min and then stained with an Alkaline Phosphatase Assay Kit (Beyotime, China). After 21 days of culture, the BMSCs were fixed and stained with Alizarin Red S. Furthermore, the osteogenic differentiation potential of BMSCs was identified by immunofluorescence staining using an anti-Osterix primary antibody (1:200).

The co-culture group and the control group were cultured in complete medium to detect the spontaneous differentiation of BMSCs in the co-culture system. After 14 days of culture, the BMSCs were identified by immunofluorescence staining with an anti-vWF antibody (1:200). Furthermore, the differentiation of BMSCs after co-culture with RAOECs was assessed by qRT-PCR and WB. The osteogenic genes were Osterix and Runx2, and the angiogenic genes were CD31 and VEGF.

### Tube formation assay

The co-culture and control groups were cultured in complete medium to assess the tube formation capability of BMSCs in the co-culture system. After 8 days of culture, the BMSCs in the two groups were digested with trypsin and seeded in 96-well plates precoated with 50 µl/well growth factor-reduced Matrigel (BD, USA) at a density of 4 × 10^4^ cells/well. The cells were incubated at 37 °C in a 5% CO_2_ atmosphere for 6 h to evaluate the formation of capillary tube-like structures. Images were captured from three different fields of view in three independent samples at 100 × magnification using an inverted microscope (Carl Zeiss, Germany). Tube formation was analyzed by the Wimasis Image Analysis service^49^.

### BMSC migration assay

The co-culture and control groups were cultured in complete medium. After 8 days of culture, the BMSCs in the two groups were digested with trypsin, and 5 × 10^4^ cells were seeded in transwell chambers in each well of a 24-well plate with 200 µl of complete medium. The transwell chamber pore size was 8 µm. The bottom chambers contained 500 µl of complete medium. After 6 h of incubation, the complete medium was removed, and 500 µl of 4% PFA were added to the bottom well for 10 min to fix the cells that had invaded through the transwell membrane; these cells were then stained with 0.1% crystal violet (Solarbio, China). Images were captured from three different fields of view in three independent samples at 50 × magnification using an inverted microscope (Carl Zeiss, Germany). Cells were counted using ImageJ software.

### Changes in signaling pathways in the co-culture system and prevascularized TEBGs

We first detected the expression levels of molecules in the RhoA/ROCK pathway in BMSCs to explore the key factors regulating the endothelial differentiation of BMSCs co-cultured with RAOECs. After 14 days of co-culture, the RhoA/ROCK signaling pathway in BMSCs was assessed by immunofluorescence staining with anti-RhoA (ab187027, 1:1000, Abcam, USA) and anti-p-RhoA (S188, ab192187, 1:1000, Abcam, USA) antibodies. Total RhoA protein including activated GTP-bound RhoA and inactivated GDP-bound RhoA. The levels of intermediates in the RhoA/ROCK pathway were analyzed using qRT-PCR and WB.

We compared the levels of RhoA expression within the different prevascularized TEBG pores located close to or far from the vascular bundle to further whether the RhoA/ROCK signaling pathway was downregulated after vascularization in vivo. After 1 week, RhoA expression was determined in the prevascularized TEBG group by immunofluorescence staining.

### Measurement of the FGF2 protein concentration by enzyme-linked immunosorbent assay (ELISA)

FGF2 concentrations in the control and co-culture groups were determined using the Rat FGF2 Quantikine ELISA Kit (R&D Systems, Minneapolis, MN, USA), according to the manufacturer’s instructions. The optical density of each well was determined within 30 min using a microplate reader (Synergy H1, BioTek, Winooski, VT, USA) by measuring the absorbance at 450 nm.

### Addition of FGF2 to the control group and an FGF receptor inhibitor to the co-culture group

To further identify the key role of FGF2 in the activation of RhoA signaling and endothelial differentiation of BMSCs, different doses of recombinant rat FGF2 (PeproTech, Rocky Hill, NJ, USA) were added to the control group every 3 days to further identify the key role of FGF2 in the inactivation of RhoA signaling and endothelial differentiation of BMSCs. The doses were 0, 5, 10 and 20 ng.

In a complementary experiment, different doses of an FGF receptor (FGFR) inhibitor (NVP-BGJ398, Selleck, Houston, TX, USA) were added to the co-culture group every 3 days. The concentrations were 0, 0.5, 2 and 5 μM.

After 7 days of culture, the levels of RhoA/ROCK signaling pathway markers and CD31 in the 8 treated groups were analyzed by qRT-PCR and WB.

### Quantitative real-time polymerase chain reaction

Total RNA was extracted from BMSCs in the control and co-culture groups using a Total RNA Kit (Omega Bio-tek, Norcross, GA, USA), according to the manufacturer’s instructions. The total RNA (500 ng) was reverse-transcribed using the Prime Script TM RT Master Mix (TaKaRa, Japan) with a PCR System 9700 thermal cycler (GeneAmp, ABi, Carlsbad, CA, USA), and the relative levels of the indicated genes were assessed by real-time PCR using SYBR Premix Ex Taq TM (TaKaRa, Japan) with a CFX96TM real-time system instrument (Bio-Rad, Hercules, CA, USA), according to the manufacturer’s protocol.

The examined genes were osteogenic genes (Osterix and Runx2), angiogenic genes (CD31 and VEGF), and signaling pathway genes (FGF2, RhoA, ROCK1 and ROCK2). The primers are reported in Table 1.

**Table 1:** Primer sequences of target genes

Gene	Forward	Reverse
Osterix	5′-TGACTGCCTGCCTAGTGTCTACA-3′;	5′-TGGATGCCCGCCTTGT-3′
RUNX2	5′-CCGATGGGACCGTGGTT-3′	5′-CAGCAGAGGCATTTCGTAGCT-3′
CD31	5′-GAACAAACTTGCAAGGAGCAGGAA-3′	5′-CACGGAGCAAGAAAGACTCTGA-3′
VEGF	5′-GCTCTCTTGGGTGCACTGGA-3′	5′-CACCGCCTTGGCTTGTCACA-3′
RhoA	5′-ACTCGGAGTCCTCGCCTTGA-3′	5′-TCTGGGAACTGGTCCTTGCTG-3′
ROCK-1	5′-CTGGACATTTGAAGTTAGCCG-3′	5′-CCAACTGCTGTATCACATCGTACC-3′
ROCK-2	5′-CCCGATCATCCCCTAGAACC-3′	5′-TTGGAGCAAGCTGTCGACTG-3′
FGF2	5′-TTCCCACCCGGCCACTTCAAG-3′	5′-GTTCGCACACACTCCCTTGA-3′
GAPDH	5′-CAGCAAGGATACTGAGAGCAAGAG-3′	5′- GGATGGAATTGTGAGGGAGATG-3′

### Western blotting

BMSCs were washed three times with PBS and lysed in RIPA lysis buffer containing 1 mM β-mercaptoethanol for 30 min on ice. The cell lysate was centrifuged at 12000 rpm for 15 min at 4 °C. The protein concentration of the supernatant was determined using a BCA kit (Beyotime, China). The supernatant was combined with protein loading buffer and boiled at 100 °C for 4 min to denature proteins. Equal amounts of protein were loaded into the wells of a 10% SDS-PAGE gel and electrophoretically separated for 2 h at 90 V. Then, proteins were transferred from the gel to PVDF membranes (Thermo Scientific, Waltham, MA, USA) and blocked with milk for 1 h at room temperature. Membranes were incubated with primary antibodies overnight at 4 °C and secondary antibodies conjugated to horseradish peroxidase (HRP, GE Healthcare, USA). The primary antibodies were anti-Runx2 (ab54868, 1:1000, Abcam, USA), anti-CD31 (1:1000), anti-RhoA (1:1000), anti-p-RhoA (1:1000) and anti-β-actin (ab8226, 1:3000, Abcam, USA). Signals were visualized using an enhanced chemiluminescence (ECL) kit (GE Healthcare, USA).

### Statistics analysis

Data are presented as the mean ± s.e.m. Comparisons between two groups were accomplished using independent sample *t*-tests and correlation analyses. All data demonstrated a normal distribution and similar variation between groups. Statistical analysis was performed using SPSS 22.0 software. *P* values < 0.05 were deemed significant, with * representing *P* < 0.05, **P < 0.01 and ***P < 0.001.
